# Obesity and Diabetes in an Arab population: Role of Adenovirus 36 Infection

**DOI:** 10.1038/s41598-020-65008-x

**Published:** 2020-05-15

**Authors:** Nader Lessan, Koramannil R. Saradalekshmi, Budour Alkaf, Maria Majeed, Maha T. Barakat, Zendra P. L. Lee, Richard L Atkinson

**Affiliations:** 1 Imperial College London Diabetes Centre, Abu Dhabi, UAE; 2 Virginia Commonwealth University, Richmond, VA USA; 3 Obetech Obesity Research Center, Richmond, VA USA

**Keywords:** Predictive markers, Epidemiology

## Abstract

Prior infection with adenovirus 36 (Adv36) has been associated with increased adiposity, improved insulin sensitivity, and a lower prevalence of diabetes. This study investigated the prevalence of Adv36 seropositivity and its association with obesity and diabetes among adults attending a diabetes centre in the UAE.Participants (N = 973) with different weight and glucose tolerance categories were recruited. Adv36 seropositivity (Adv36 + ) was assessed using ELISA. Differences among groups were analyzed using statistical tests as appropriate to the data. Prevalence of Adv36+ in the study population was 47%, with no significant difference in obese and non-obese subgroups (42.5% vs 49.6% respectively; p=non-significant). Females were more likely to be Adv36+ compared to males (odds ratio 1.78; 95% CI 1.36–2.32, p < 0.001). We found no significant association between Adv36 seropositivity and different BMI categories, or glucose tolerance status. In our population, the effect of Adv36 infection on lipid profile varied between healthy individuals and individuals with obesity. Adv36 infection is more prevalent in the UAE than in other countries but has no association with obesity. Our study found that females were more likely to be Adv36 positive regardless of weight or diabetes status.

## Introduction

Obesity is the fifth leading risk factor for global deaths, and promotes the development of several chronic illnesses, including cardiovascular diseases, cancer, and diabetes^1^. The global obesity prevalence has increased dramatically over the last 40 years, from 3.2% in 1975 to 10.8% in 2014 in men, and from 6.4% to 14.9% in women. In the Middle East and North Africa (MENA) region, obesity prevalence surpassed 30% in 2014. The largest share of the world’s population with severe obesity in 2014 was in high income English-speaking countries (27.1%; 50 million), followed by 13.9% (26 million) in the MENA region^2^. Furthermore, according to the global burden of disease (GBD) study^3^, the MENA region had the second highest prevalence of obesity in women of 33.9% in 2013. The majority of the countries within the MENA region are among those with the highest rates of obesity worldwide^3^.

Studies investigating the reasons underlying the epidemic of obesity in the MENA region have mainly focused on the role of sociocultural variables including dietary, lifestyle and physical activity in addition to hereditary factors. Along with the dramatic rise in obesity has come a rapid rise in diabetes in the MENA region, which now has the second highest prevalence in the world^4^.

Over the last 20 years, there has been accumulating evidence supporting the hypothesis that viral infections may be associated with obesity in animals and humans. Eight infectious agents, including canine distemper virus, Rous associated virus, type 7 (RAV-7), Borna virus, scrapie agents, SMAM-1 avian adenovirus, human adenovirus-5, human adenovirus-37, and human adenovirus-36 (Adv36) have been implicated in contributing to obesity in animals and humans^5^. Adenoviruses are the only infectious agents reported to be linked with adiposity in both experimental animal models and human studies^6^.

Three human adenoviruses have been related to obesity, with adenovirus 36 being the most studied serotype^7–9^. Bioinformatics comparisons have identified significant differences between Adv36 and other human adenoviruses, suggesting unique functions of Adv36 that possibly can be linked with adipose tissue^10^.

In humans, the correlation of natural Adv36 infection with the development of obesity has been implicated in several studies across different ethnic populations, in both adults and children. Association of Adv36 infection with obesity in humans was first reported from a US population^11^. Although a few studies have been inconsistent with these findings^12–14^, many others in multiple ethnic populations, including North Americans^15–18^, Mexico^19^, Europeans^20–24^, Turkey^25,26^ and East Asians^27,28^, Chileans^29^ have confirmed them. The prevalence of adenovirus seropositivity differed across ethnic groups, with an average prevalence ranging from 65% in Italy to 6% in Belgium/Holland^13^.

The association of Adv36 with diabetes is more controversial. *In vitro* studies show Adv36 enhances glucose transport into cells and improves insulin sensitivity. Animal studies also show that Adv36 infection improves glucose tolerance and tends to lower insulin levels^30,31^. Some human studies indicate a decreased prevalence of diabetes in individuals with Adv36 antibodies^28,32^, but others have suggested that prior Adv36 infection is associated with a higher prevalence of diabetes^33^.

In 2013, the UAE was estimated to have an obesity prevalence of 29% among adults, and was ranked number 21 worldwide^3^ and to have a prevalence of diabetes of 15.58% in 2017^34^. In this study, we aimed to investigate the role of Adv36 infection in the epidemic of obesity and diabetes in this population by: (1) identifying the prevalence of Adv36 seropositivity among adults living in the UAE; and (2) assessing the association of Adv36 with obesity and diabetes in the population.

## Results

### Study population characteristics and prevalence of Adenovirus 36 seropositivity

Characteristics of the population studied are summarized in Table 1. Ninety percent (N = 875) of the study population were Emiratis (natives of the United Arab Emirates) and the remaining were expatriates, predominantly of Arab ethnicity. Among the 973 adults who participated in the study, 458 (47.1%) were found to be seropositive for Adv36 while 515 (52.9%) were seronegative. The sex distribution among the participants was comparable with a male to female ratio of 0.96. The prevalence of obesity (BMI ≥ 30 kg/m2) in this population was 35.8%; a further 35.6% were overweight (BMI ≥ 25 and ≤29.9 kg/m^2^) and 28.6% had a normal weight (BMI ≤ 24.9 kg/m^2^).

**Table 1:** Characteristics of the study population.

Characteristics	Sub group	N (%)
N	Male	477 (49.1)
	Female	496 (50.9)
	Total	973
Adenovirus 36	Seropositive	458 (47.1)
	Seronegative	515 (52.9)
BMI (kg/m^2^)	<24.9 (Normal weight)	278 (28.6)
	25–29.9(Overweight)	346 (35.6)
	>30 (Obese)	349 (35.8)
**Characteristics**	**Sub-group**	**Mean** ± **SD**
Age (years)	Male	43.3 ± 13.8
	Female	42.4 ± 12.9
	All Subjects	42.9 ± 13.4
Waist circumference (cm)	All subjects	95.8 ± 12.8
Waist to hip ratio	All Subjects	0.9 ± 0.08
Blood Pressure (mmHg)	Systolic	120.2 ± 16.8
	Diastolic	71.3 ± 21.7
Lipid Profile (mmol/l)	HDL-c	1.3 ± 0.4
	LDL-c	2.9 ± 0.9
	TC	4.5 ± 1.0
	TG	1.4 ± 0.9
Glycaemic Control	HbA1C (%)	6.6 ± 1.6

BMI: Body mass index, LDL-c: Low Density Lipoprotein cholesterol, HDL-c: High Density Lipoprotein cholesterol, TC: Total Cholesterol, TG: Triglycerides, HbA1c: Glycosylated Haemoglobin.

### Comparison of Adv36 seropositive and seronegative participants

Comparison of mean BMI, HbA1c, lipid profile, and body composition between seropositive and seronegative individuals are summarized in Table 2. After adjusting for age, gender and BMI, there was no significant difference in clinical and anthropological parameters between Adv36+ and Adv36- groups. Other clinical parameters such as Blood pressure, Liver enzymes and haemoglobin were also compared between the two groups (Supplementary Table 1).

**Table 2:** Difference in clinical and anthropometric parameters between Adv36 seropositive and seronegative individuals.

	Overall (N = 973)			Males (N = 477)			Females (N = 496)		
	ADV 36 (+)	ADV 36 (-)	*P* value	ADV 36 (+)	ADV 36 (-)	*P* value	ADV 36 (+)	ADV 36 (-)	*P* value
N	458	515		197	280		261	235	
**Anthropometry**									
Age (yrs.)	42.2 ± 13.9	42.6 ± 14.0	0.622^a^	41.9 ± 14.3	43.6 ± 14.3	0.199^a^	42.4 ± 13.7	41.4 ± 13.7	0.439^a^
Weight (kg)	76.2 ± 17.0	79.0 ± 16.3	0.09	82.0 ± 15.9	82.5 ± 16.4	0.71	71.8 ± 16.5	74.8 ± 15.1	0.26
BMI (kg/m^2^)	28.5 ± 5.7	28.9 ± 5.6	0.158	27.8 ± 4.7	28.0 ± 5.0	0.539	29.0 ± 6.3	29.8 ± 6.0	0.148
Waist (cm)	95.2 ± 13.5	96.3 ± 12.0	0.427	98.7 ± 12.2	98.2 ± 12.0	0.117	91.8 ± 13.5	94.1 ± 12.0	0.067
WHR	0.9 ± 0.08	0.9 ± 0.07	0.284	1.0 ± 0.0	1.0 ± 0.06	0.104	0.9 ± 0.08	0.9 ± 0.07	0.48
**Body composition**									
Fat (%)	31.3 ± 9.5	30.7 ± 9.3	0.67	25.1 ± 7.3	25.4 ± 7.0	0.957	36.3 ± 7.9	37 ± 7.5	0.236
Fat Mass (kg)	25.0 ± 11.4	25.5 ± 10.8	0.178	21.5 ± 10.1	22.2 ± 10.7	0.588	27.3 ± 11.7	28.7 ± 10.7	0.147
Fat Free mass (kg)	52.4 ± 10.8	54.3 ± 10.3	0.365	60.9 ± 8.7	60.7 ± 8.7	0.617	44.8 ± 5.7	46.1 ± 5.9	0.03
**Lipid profile**									
HDL-c (mmol/L)	1.3 ± 0.4	1.3 ± 0.3	0.156	1.2 ± 0.3	1.2 ± 0.3	0.793	1.5 ± 0.3	1.4 ± 0.3	0.038
LDL-c (mmol/L)	2.9 ± 0.9	2.9 ± 0.8	0.308	2.8 ± 1.0	2.9 ± 0.9	0.127	2.9 ± 0.8	2.9 ± 0.8	0.993
TC (mmol/L)	4.4 ± 1.0	4.5 ± 0.9	0.354	4.3 ± 1.0	4.4 ± 1.0	0.163	4.6 ± 0.9	4.6 ± 0.8	0.961
TG (mmol/L)	1.3 ± 0.8	1.4 ± 0.9	0.704	1.4 ± 0.9	1.4 ± 0.9	0.766	1.2 ± 0.6	1.3 ± 0.9	0.205
**Glycaemic profile**									
HbA1c (%)	6.7 ± 1.8	6.5 ± 1.7	0.055^b^	6.9 ± 1.9	6.7 ± 1.6	0.2	6.5 ± 1.7	6.2 ± 1.6	0.037

ANCOVA with age, gender and BMI as covariates. BMI: Body mass index, WHR: Waist to Hip Ratio, HDL-c: High Density Lipoprotein cholesterol, LDL-c: Low Density Lipoprotein cholesterol, TC: Total Cholesterol, TG: Triglycerides, HbA1c: Glycated Haemoglobin. a Unpaired t test, b Mann - Whitney U test.

### Adv36 seropositivity and its associations

#### Gender

**Figure 1:**
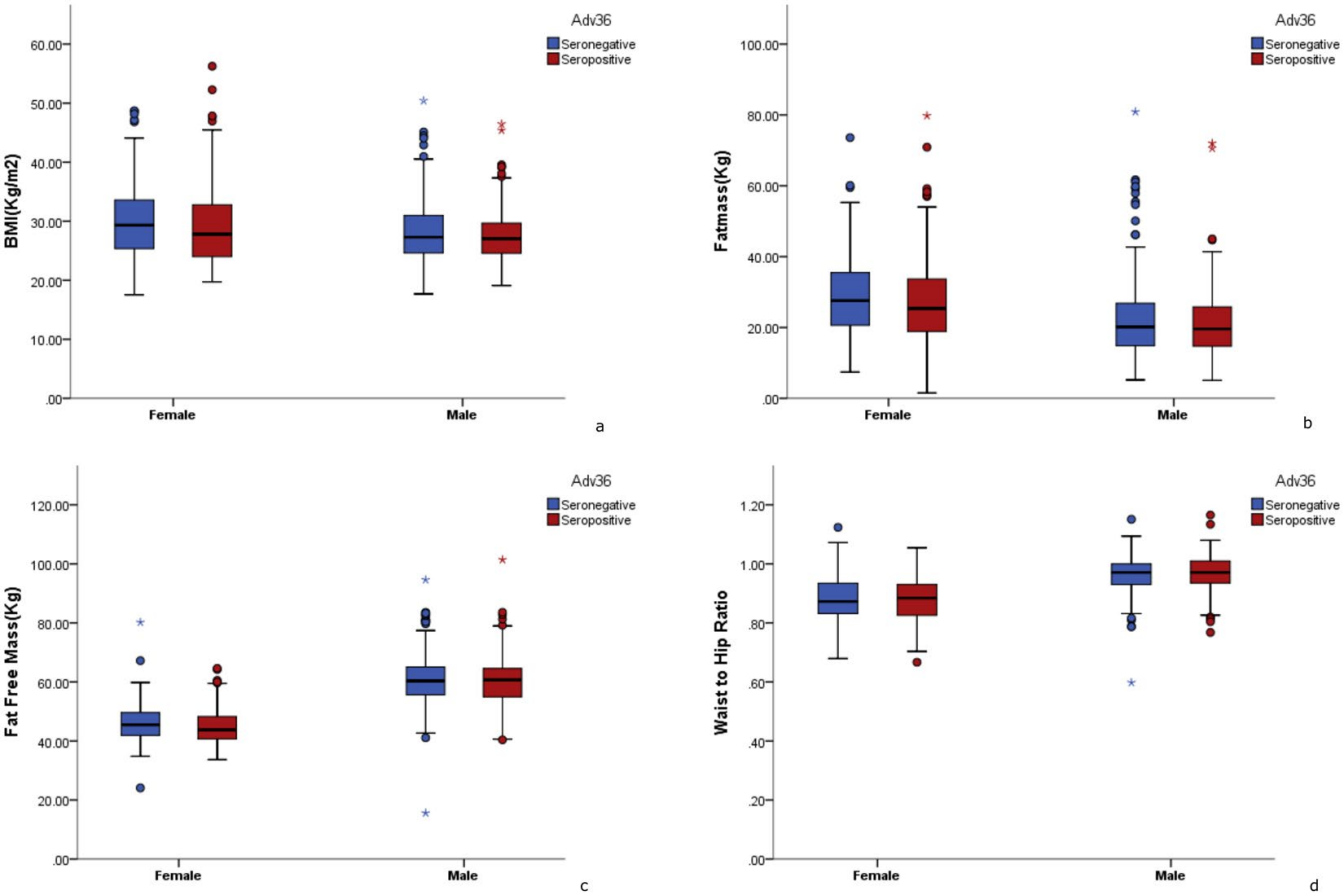
Body composition analysis in females and males based on Adv36 status. Blue bars represent Adv36 seronegatives, red bars represent Adv36 seropositives1) Body mass index by sex and Adv36 status 2) Fat mass by sex and Adv36 status, 3) Fat free mass by sex and Adv36 status, 4) Waist to hip ratio by sex and Adv36 status.

Prevalence of Adv36 seropositivity was significantly higher in women (53%) compared to men (41%) and in general, women had increased likelihood of being Adv36 + (OR 1.78; 95% CI 1.36–2.32, p < 0.001) (Table 3). There was no difference in studied parameters between Adv36 positive and negative men (Fig. 1). However, in women, seropositivity was associated with decreased fat free mass (*p* = 0.030), increased HDL-c (*p* = 0.038) and increased HbA1c (*p* = 0.037) (Table 2).

**Table 3:** Association of Adv 36 seropositivity with gender, obesity and diabetes.

	N	ADV 36 + (N %)	Odds Ratio (95% CI)	P value
**Gender**				
Male	477	197 (41.3)	Ref.	
Female	496	261 (52.6)	1.78 (1.36–2.32)	<0.001
**Obesity** ^**a**^				
Non - obese (BMI ≤ 29.9)	625	310(49.6)	Ref.	
Obese (BMI ≥ 30)	348	148(42.5)	0.696 (0.532–0.912)	0.034
**BMI Categories** ^**b**^				
Normal Weight (BMI ≤ 24.9)	279	140 (50.4)	Ref.	
Overweight (BMI 25–29.9)	346	169 (48.8)	0.98 (0.71–134)	0.889
Obese Class I (BMI 30–34.9)	221	87 (39.7)	0.61 (0.42–0.87)	0.007
Obese Class II (BMI 35–39.9)	84	42 (48.8)	0.95 (0.58–1.55)	0.834
Obese Class III (BMI ≥ 40)	43	20 (45.4)	0.74 (0.39–1.56)	0.546
**Glycaemic Status** ^**c**^				
Normal Glucose Tolerance	301	135 (44.8)	Ref.	
Type 1 Diabetes	181	94 (52.0)	1.41 (0.97–2.08)	0.073
Type 2 Diabetes	289	141 (48.8)	1.45 (0.99–2.13)	0.055
Prediabetes	202	88 (43.5)	1.07 (0.74–1.57)	0.702

Logistic regression analysis with Adv36 status (indicator): seropositive and seronegative (seronegative as reference).

^a^Binary logistic regression analysis; Covariates: Age in years and Gender.

^b^Multinomial logistic regression analysis; Covariates: Age in years and Gender.

^c^Multinomial logistic regression analysis; Covariates: Age in years, Gender and BMI.

#### Obesity

The prevalence of seropositivity was higher (49.6%) in subjects who were not obese compared to subjects with obesity (42.5%). Seropositivity was found to be negatively correlated with obesity [obese (BMI ≥ 30 kg/m2) vs non-obese (BMI ≤ 29.9 kg/m2);(β = −0.362; OR = 0.696 (0.532–0.912); Pearson Chi-Square *p* = 0.034)]. There was a negative association between BMI categories and Adv36+ overall, and only Class I obesity had statistically significant association with Adv36 + (crude OR 0.61; 95%CI 0.42–0.987, p = 0.007) (Table 3). Adenovirus seropositivity was significantly associated with lower LDL cholesterol (p = 0.013), total cholesterol (p = 0.009) and triglycerides (p = 0.007) in healthy weight individuals (N = 279). In the subgroup with obesity, Adv36 seropositive individuals had significantly higher total cholesterol (p = 0.023; Table 4).

**Table 4:** Difference in clinical and anthropometric characteristics between Adv36 positive and negative groups among subgroups based on BMI.

	Normal weight (N = 279)			Overweight (N = 346)			Obese (N = 348)		
	ADV 36 (+)	ADV 36 (-)	*P* value	ADV 36 (+)	ADV 36 (-)	*P* value	ADV 36 (+)	ADV 36 (-)	*P* value
N	141	138		169	177		148	200	
**Anthropometry**									
Age (years)	39.9 ± 14.5	41.2 ± 15.7	0.485^a^	44.2 ± 13.9	43.5 ± 14.1	0.646^a^	41.9 ± 13.3	42.8 ± 12.9	0.514^a^
Weight (kg)	60.4 ± 8.2	63.5 ± 8.9	0.239	74.8 ± 9.3	75.6 ± 8.3	0.923	91.9 ± 15.6	92.5 ± 14.7	0.618
BMI (kg/m^2^)	22.8 ± 1.4	22.7 ± 1.7	0.718	27.4 ± 1.5	27.3 ± 1.4	0.607	34.9 ± 4.8	34.5 ± 4.2	0.455
Waist (cm)	83.3 ± 9.8	85.5 ± 8.9	0.541	93.6 ± 8.9	94.6 ± 7.7	0.76	105.3 ± 12.1	104.5 ± 11.1	0.145
WHR	0.9 ± 0.08	0.9 ± 0.09	0.654	0.9 ± 0.08	0.9 ± 0.07	0.56	0.9 ± 0.08	0.9 ± 0.08	0.221
**Body composition**									
Fat (%)	23.8 ± 7.5	22.4 ± 6.9	0.513	29.9 ± 6.9	29.4 ± 7.07	0.983	39.2 ± 7.6	37.6 ± 7.2	0.716
Fat Mass (kg)	14.3 ± 4.6	15.1 ± 8.3	0.056	22.3 ± 5.1	22.0 ± 4.76	0.991	36.3 ± 10.6	34.8 ± 9.3	0.897
Fat Free mass (kg)	46.3 ± 8.6	49.4 ± 8.7	0.208	52.9 ± 10.1	53.5 ± 9.71	0.322	55.8 ± 11.3	57.6 ± 11.1	0.449
**Lipid profile**									
HDL -c (mmol/L)	1.5 ± 0.5	1.4 ± 0.4	0.246	1.3 ± 0.4	1.3 ± 0.4	0.719	1.3 ± 0.4	1.2 ± 0.3	0.08
LDL-c (mmol/L)	2.6 ± 0.9	2.9 ± 0.9	0.013	2.8 ± 0.8	2.9 ± 0.8	0.254	3.1 ± 0.8	2.9 ± 0.9	0.052
TC (mmol/L)	4.3 ± 1.0	4.6 ± 0.9	0.009	4.4 ± 0.9	4.5 ± 0.9	0.198	4.7 ± 0.9	4.4 ± 1.0	0.023
TG (mmol/L)	1.0 ± 0.6	1.3 ± 0.9	0.007^b^	1.4 ± 0.9	1.3 ± 0.9	0.267	1.4 ± 0.8	1.5 ± 0.9	0.854
**Glycaemic profile**									
HbA1c (%)	6.6 ± 1.8	6.8 ± 2.0	0.689	6.7 ± 1.8	6.5 ± 1.6	0.164	6.8 ± 1.8	6.3 ± 1.5	0.019^b^

ANCOVA with age, gender and BMI as covariates. BMI: Body Mass Index, WHR: Waist to Hip Ratio, HDL-c: High Density Lipoprotein cholesterol, LDL: Low Density Lipoprotein cholesterol, TC: Total Cholesterol, TG: Triglycerides, HbA1c: Glycated Haemoglobin.

a Unpaired t test, b Mann - Whitney U test.

#### Diabetes status

Type 1 diabetes, type 2 diabetes and prediabetes showed no significant association with Adv 36 seropositivity (Table 3). There were no differences in studied parameters between Adv36 seropositive and seronegative individuals in different diabetes subgroups and with the normal glucose tolerance group (Table 5). In type 1 diabetes group, there was no difference in mean Glutamic Acid Decarboxylase (GAD) antibody titer between ADV 36 seropositive and seronegative individuals (p = 0.272). However, when individuals with type 1 diabetes were stratified into GAD negative, low positive and positive subgroups, Adv36 seropositivity significantly correlated with high GAD antibody titer (Spearman p = 0.036) (Supplementary Table 2).

**Table 5:** Difference in clinical and anthropometric characteristics between Adv36 positive and negative groups among subgroups based on glycaemic status.

	NGT (N = 301)			Prediabetes(N = 202)			Type 1(N = 181)			Type 2 (N = 289)		
	ADV 36 (+)	ADV 36 (-)	*P* value	ADV 36 (+)	ADV 36 (-)	*P* value	ADV 36 (+)	ADV 36 (-)	*P* value	ADV 36 (+)	ADV 36 (-)	*P* value
N	135	166		88	104		94	87		141	148	
**Anthropometry**												
Age (yrs.)	36.7 ± 11.3	37.8 ± 11.5	0.399^a^	43.9 ± 9.8	43.4 ± 12.3	0.750^a^	31.6 ± 10.0	33.2 ± 12.3	0.327^a^	53.8 ± 11.4	53.5 ± 10.8	0.864^a^
Weight (kg)	73.2 ± 19.9	76.7 ± 1.3	0.24	81.0 ± 17.8	84.8 ± 16.2	0.266	74.3 ± 17.4	76.0 ± 14.0	0.808	77.4 ± 17.2	78.6 ± 15.2	0.875
BMI (kg/m^2^)	27.6 ± 5.2	28.4 ± 5.9	0.247	30.4 ± 6.5	30.8 ± 5.4	0.288	27.3 ± 5.0	27.2 ± 4.7	0.973	28.9 ± 5.8	28.7 ± 5.4	0.967
Waist (cm)	93.8 ± 11.6	94.9 ± 13.0	0.063	95.3 ± 11.3	98.9 ± 10.9	0.11	89.8 ± 14.8	91.6 ± 11.7	0.411	99.1 ± 12.3	98.8 ± 11.7	0.425
WHR	0.8 ± 0.08	0.9 ± 0.08	0.098	0.9 ± 0.0	0.9 ± 0.06	0.101	0.8 ± 0.0	0.9 ± 0.07	0.412	0.9 ± 0.06	0.9 ± 0.0	0.087
**Body composition**												
Fat (%)	31.0 ± 9.3	30.4 ± 9.4	0.564	34.0 ± 9.7	33.1 ± 9.0	0.706	28.2 ± 9.0	28.0 ± 9.0	0.218	32.0 ± 9.2	30.7 ± 9.1	0.832
Fat Mass (kg)	23.4 ± 10.0	24.5 ± 12.1	0.527	26.6 ± 12.8	28.2 ± 11.6	0.692	21.6 ± 9.9	21.9 ± 9.5	0.142	25.4 ± 11.8	24.9 ± 9.9	0.353
Fat Free mass (kg)	50.4 ± 9.3	52.6 ± 10.7	0.745	53.1 ± 10.9	56.1 ± 10.4	0.841	53.0 ± 12.2	54.3 ± 8.8	0.905	52.0 ± 10.7	53.6 ± 10.9	0.169^b^
**Lipid profile**												
HDL-c (mmol/L)	1.4 ± 0.4	1.3 ± 0.3	0.304	1.3 ± 0.3	1.2 ± 0.2	0.057	1.4 ± 0.4	1.4 ± 0.4	0.562	1.2 ± 0.4	1.2 ± 0.4	0.464
LDL-c (mmol/L)	2.9 ± 0.8	3.0 ± 0.8	0.779	3.2 ± 0.8	3.1 ± 0.8	0.471	2.8 ± 0.8	2.7 ± 0.8	0.921	2.5 ± 0.8	2.6 ± 0.9	0.325
TC (mmol/L)	4.6 ± 0.9	4.6 ± 0.8	0.888	4.8 ± 0.9	4.7 ± 0.9	0.397	4.4 ± 0.9	4.4 ± 0.9	0.673	4. 1 ± 0.9	4.2 ± 1.0	0.232
TG (mmol/L)	1.1 ± 0.8	1.1 ± 0.7	0.362	1.3 ± 0.7	1.5 ± 0.9	0.365	1.1 ± 0.7	1.1 ± 0.5	0.874	1.6 ± 0.9	1.7 ± 1.1	0.475
**Glycaemic profile**												
HbA1c (%)	5.1 ± 0.6	5.2 ± 0.6	0.897	5.5 ± 0.4	5.5 ± 0.4	0.454	8.3 ± 1.7	8.2 ± 1.7	0.676	7.5 ± 1.6	7.2 ± 1.6	0.202

ANCOVA with age, gender and BMI as covariates. NGT: Normal Glucose Tolerance, BMI: Body Mass Index, WHR: Waist to Hip Ratio, HDL-c: High Density Lipoprotein cholesterol, LDL: Low Density Lipoprotein cholesterol, TC: Total Cholesterol, TG: Triglycerides, HbA1C: Glycated Haemoglobin.

a Unpaired t test, b Mann - Whitney U test.

## Discussion

Obesity and type 2 diabetes are highly prevalent in the Middle East. The rapid increased prevalence in the Gulf region in the last few decades is generally attributed to urbanisation and the accompanying unfavourable lifestyle changes. While there are no doubts about the drastic change in lifestyle in this region, whether the physical lifestyle change alone can be responsible for a phenomenon rightly termed an epidemic is a matter of debate.

Epidemics are often attributable to infectious organisms including viruses. It is thus not surprising that a virus that has been proven as a causative agent of obesity. *In vivo* infection with Adv36 in primates^35^, rodents, and chickens^30^ have shown increases in body fat and weight gain. Meta analyses suggest association of Adv36 infection with risk of obesity and weight gain^36,37^.

In the current study, we found around 47% Adenovirus 36 seropositivity prevalence in the UAE population. This prevalence is similar to that reported from Iran^38^ and Italy^22,23^, but higher than that reported from US^11^, South Korea^27^ and Sweden^21^, and lower than Mexico^19^. To our knowledge, ours is the first such study in an Arab population. Similar to studies from Iran^38^ and China^39^, and in contrast to several other studies, in our population, we found no correlation between Adv36 seropositivity with obesity in spite of its the high prevalence.

Apart from having an effect on fat accumulation and weight gain, Adv36 infection has been reported to result in metabolic changes including alterations in lipid profile. The mechanisms of altered lipid profile in Adv 36 seropositive individuals remains unclear^11^.The reports are inconsistent and range from favourable effect on the serum lipid profile in Adults in US population^11^ to an undesirable increase in LDL cholesterol, total cholesterol and triglycerides and a decrease in HDL cholesterol in other populations^20^. In our population, the effect of Adv36 infection on lipid profile varied between healthy individuals and individuals with obesity. Among individuals with healthy BMI ( ≤ 24.9), decreased serum levels of LDL cholesterol, total cholesterol and triglycerides were observed in Adv36 positive individuals. However, Adv36 infection in individuals with obesity was found to be associated with increased HDL, LDL and Total cholesterol in the population we studied. The protective or favourable effect of Adv 36 seropositivity on the lipid profile might be masked by obesity in this group.

Other effects of Adv 36 infection include changes in insulin sensitivity and glucose tolerance status. Adv36 infection has been linked to enhanced insulin sensitivity and glucose uptake *in vitro*^8,30,40–42^ and improved glucose tolerance and/or insulin sensitivity in humans and animals^28,31–33^. Interestingly, we did not find an improved glucose tolerance in Adv36 seropositive subjects in our study. Indeed, we found a significantly higher HbA1c in Adv36 seropositive patients with obesity and type 2 diabetes, p = 0.004 (Supplementary Table 3). Viral infections are known to trigger autoimmunity in pancreatic beta cells and further clinical presentation of type 1 diabetes^43^. However, association of Adv36 infection with GAD antibodies has not been reported so far. In our patients with type 1 diabetes, Adenovirus 36 seropositivity was associated a high GAD antibody titer suggesting that adenovirus infection could be a trigger for autoimmunity in pancreatic beta cells.

Establishing a causative role for an infective organism in human obesity is challenging for ethical reasons. Population studies such as the current study may offer support to such hypotheses. However, results from different studies have been inconsistent and reported association with obesity and diabetes has been variable. It may be that for Adv36 exposure to result in obesity humans the presence of some other predisposition such as racial or genetic background is needed.

Another possibility for the variation in Adv36 prevalence in different populations may be methodological. Serum neutralizing assay and ELISA are broadly used to measure the presence of ADV36 infection. If a given ELISA assay is insufficiently specific, it will pick up antibodies against other adenoviruses and dilute the Adv36-obesity correlation. This has been shown for some ELISAs in comparison to the serum neutralization assay^44^. However, the serum neutralization assay is significantly less sensitive than our ELISA assay. In a study from Sweden, we reported that the ELISA scored positive for 36.9% of the Adv36-SNA-seronegative samples^24^. Additional studies in our lab comparing known highly Adv36 positive samples serially diluted showed that the ELISA was 2–4 fold more sensitive than serum neutralization. Furthermore, this ELISA assay was tested against other animal and human adenovirus antibodies and found that there is little or no cross-reaction confirming the specificity of the test for Adenovirus 36 (unpublished data).

Finally, the absence of correlation of Adv36 seropositivity and obesity in our current study and in some other more recent studies^38,39^ as compared to the fairly strong correlations in earlier reports, suggests changes in the virus, or the environment. However, Na *et al*. showed that Adv36 was very stable across three decades with essentially no mutation^45^. It is noteworthy that the more recent studies with higher Adv36 seropositivity have been conducted mostly in the Asian populations^38,39^. Potentially, other changing environmental factors may be of importance in predisposing Adv36 positive individuals to the effects of the virus on adipose tissue^46^. It is also possible that as with many other viral infections, Adv36 antibody titers decrease over time to levels below the value for “positivity”, and thus diluting an otherwise expected association with obesity.

In conclusion, we have identified a high overall prevalence of Adv36 seropositivity, but no apparent association with weight in an Arab Middle Eastern population with a rapid rise in obesity and diabetes prevalence over a relatively short period. We have also shown a hitherto unreported finding of a higher GAD titer in Adv 36 seropositive patients with type 1 diabetes and also found Adv36 seropositivity to be associated with worse glycaemic control in women and people with obesity. The findings of this study are somewhat different from those reported in Europe and America and these findings merit confirmation in other populations.

## Methods

### Study population

The study was conducted at Imperial College London Diabetes Centre (ICLDC), Abu Dhabi. The ICLDC is a large out-patient facility, offering medical care to patients with diabetes, obesity, endocrine and general medical conditions. Participants were recruited with a pre-defined target population with the aim of achieving comparable number of subjects from different BMI classes and glucose tolerance. The study subjects were recruited from among patients visiting the clinic during their regular visits. Written informed consent was obtained from all participants at the time of recruitment. The study was approved by the Research Ethics Committee at ICLDC and followed the Declaration of Helsinki, 1996.

### Anthropometric measures and clinical examination

All participants were examined by trained nurses at ICLDC. Anthropometric measures were made, and included weight, height, waist and hip circumference and blood pressure. Height was measured to the nearest 0.5 cm, and body weight was measured to the nearest 100 g. Waist circumference was measured at the midpoint between ribs and iliac crest, and hip circumference was measured at the greater trochanters. Waist-to-hip ratio was calculated as waist circumference (cm) divided by hip circumference (cm). Body composition including BMI, fat percentage, fat mass and fat free mass were analysed using bio electric impedance analyzer (SECA, Hamburg, Germany). Obesity was defined as BMI ≥ 30 kg/m^2^. Relevant clinical data were extracted from patients’ electronic records at time of recruitment, and included age of onset of obesity and/or diabetes, family history of obesity and/or diabetes, complications of obesity and/or diabetes, and smoking history. Alcohol intake was not recorded as consumption in the population studied is known to be rare. Classification of diabetes type was based on American Diabetes Association guidelines 2019.

### Laboratory investigations

Blood samples were collected from participants following an overnight fast. HbA1c, haemoglobin and Lipid profile were analysed as part of routine laboratory investigations and the results were extracted from the electronic records. Presence of Adv36 antibodies in serum were assayed using the competitive enzyme-linked immunosorbent assay method (ELISA-Obetech Laboratories, Richmond VA)^21,24^. Samples were sent in batches and were anonymized to maintain confidentiality and blinding. Results of the assays were sent to the principal investigator along with the ELISA cut off values. Based on the cut-off values in each ELISA assay, samples were coded seropositive or seronegative.

### Statistical analyses

Baseline characteristics of the study population were analysed for frequency and mean distribution using descriptive statistics function. Homogeneity of variable distributions were tested using Levene’s test^47^. Binary logistic regression analysis on Adv 36 seropositivity was performed between individuals with and without obesity with age and gender as covariates. Differences in characteristics between Adv36 seropositive and seronegative groups were analysed using unpaired t test and ANCOVA with age, gender and BMI as covariates for normally distributed variables and Mann - Whitney U test for non-normally distributed variables. Differences in characteristics based on Adv36 status were analysed in males and females separately to avoid sex bias. The variables were transformed using natural logarithm to normalise the data and were analysed for differences in means in seropositive and seronegative groups. The sample population was further stratified into groups based on BMI (Healthy, Overweight, Obese I, Obese II and Obese III) and Glycaemic status (Normal, Prediabetic, Type 1 and Type 2) and analysed for differences in characteristics between Adv36 positive and negative subjects. Multinomial logistic regression on Adv36 seropositivity among different BMI groups and Glycaemic statuses were also performed with the healthy group and normal glucose tolerance group as references respectively. All analyses were performed using SPSS version 22 (IBM, Chicago, IL, USA).

## Supplementary information

Supplementary information.
