# Supplementary material for: Obesity and Diabetes in an Arab population: Role of Adenovirus 36 Infection
Source: Sci Rep. 2020 May 15;10:8107. doi: 10.1038/s41598-020-65008-x (PMC7229214; doi:10.1038/s41598-020-65008-x)
Supplement: Supplementary file 1 — Supplementary information. [file 41598_2020_65008_MOESM1_ESM.docx]

Obesity and Diabetes in an Arab population: Role of Adenovirus 36 Infection

Nader Lessan^1^, Koramannil R Saradalekshmi^1^, Budour Alkaf^1^, Maria Majeed^1^, Maha T Barakat^1^, Zendra P.L. Lee^3^, Richard L Atkinson^2, 3^

1. Imperial College London Diabetes Centre, Abu Dhabi, UAE
2. Virginia Commonwealth University, Richmond, VA, USA
3. Obetech Obesity Research Center, Richmond, VA, USA

Correspondence to:

Nader Lessan MD, FRCP

Email: [nlessan@icldc.ae](mailto:nlessan@icldc.ae)

**Supplementary Tables**

**Supplementary Table 1: Comparison of other clinical parameters between Adv36 seropositive and seronegative subjects**

|  | **Overall (N=973)** | |  |
| --- | --- | --- | --- |
| **Parameter** | **ADV 36 (+)** | **ADV 36 (-)** | ***P* value** |
| **N** | **458** | **515** |  |
| **ALT** | 23.6 ± 13.9 | 25.3 ± 17.6 | 0.66 |
| **AST** | 20.0 ± 9.8 | 20.5 ± 9.7 | 0.47 |
| **Haemoglobin (g/L)** | 131.0 ± 16.5 | 133.7 ± 16.9 | 0.89 |
| **SBP (mmHg)** | 121.7 ± 17.2 | 123.7 ± 17.5 | 0.83 |
| **DBP (mmHg)** | 70.6 ± 9.3 | 72.5 ± 10.8 | 0.30 |
| ANCOVA with Age Gender and BMI as covariates. ALT: Alanine Transaminase, AST: Aspartate Transaminase, SBP: Systolic Blood Pressure, DBP: Diastolic Blood Pressure | | | |

**Supplementary Table 2: Correlation of GAD antibody status and Adv36 seropositivity in Type 1 diabetic patients**

|  | **Negative** | **Low Positive** | **Positive** | **Chi Sq. P value** | **Spearman’s R** | **Spearman’s P value** |
| --- | --- | --- | --- | --- | --- | --- |
| **Adv36 (-)** | 25 | 18 | 32 | 0.001 | 0.167 | 0.036 |
| **Adv36 (+)** | 24 | 4 | 53 |  |  |  |

Negative: <5 U/ml, Low Positive: 5-30 U/ml, Positive: >30 U/ml

**Supplementary Table 3: Comparison of HbA1c levels among Adv36 seropositive and seronegative in Type 2 diabetic patients with obesity**

|  | **Adv36 (-)** | **Adv36 (+)** | **P value** |
| --- | --- | --- | --- |
| **N** | 54 | 43 |  |
| **HbA1c (%)** | 6.8 ± 1.3 | 7.7 ± 1.6 | 0.004 |

Unpaired T test (Mean ± SD)
